# Influence of Genes Suppressing Interferon Effects in Peripheral Blood Mononuclear Cells during Triple Antiviral Therapy for Chronic Hepatitis C

**DOI:** 10.1371/journal.pone.0118000

**Published:** 2015-02-23

**Authors:** Sayuki Iijima, Kentaro Matsuura, Tsunamasa Watanabe, Koji Onomoto, Takashi Fujita, Kyoko Ito, Etsuko Iio, Tomokatsu Miyaki, Kei Fujiwara, Noboru Shinkai, Atsunori Kusakabe, Mio Endo, Shunsuke Nojiri, Takashi Joh, Yasuhito Tanaka

**Affiliations:** 1 Department of Virology and Liver Unit, Nagoya City University Graduate School of Medical Sciences, Nagoya, Japan; 2 Department of Gastroenterology and Metabolism, Nagoya City University Graduate School of Medical Sciences, Nagoya, Japan; 3 Infectious Disease and Immunogenetics Section, Department of Transfusion Medicine, Clinical Center, National Institutes of Health, Bethesda, MD, United States of America; 4 Laboratory of Molecular Genetics, Institute for Virus Research, Kyoto University, Kyoto, Japan; 5 Division of Internal Medicine, Toyokawa City Hospital, Toyokawa, Japan; 6 Division of Gastroenterology, Nagoya Daini Red Cross Hospital, Nagoya, Japan; Kaohsiung Medical University Hospital, Kaohsiung Medical University, TAIWAN

## Abstract

The levels of expression of interferon-stimulated genes (ISGs) in liver are associated with response to treatment with pegylated interferon (PEG-IFN) plus ribavirin (RBV). However, associations between the responses of ISGs to IFN-based therapy and treatment efficacy or interleukin-28B (*IL28B*) genotype have not yet been determined. Therefore, we investigated the early responses of ISGs and interferon-lambdas (IFN-λs) in peripheral blood mononuclear cells (PBMCs) during PEG-IFN/RBV plus NS3/4 protease inhibitor (PI) therapy. We prospectively enrolled 50 chronic hepatitis C patients with HCV genotype 1, and collected PBMCs at baseline, 8 and 24 h after the initial administration of PEG-IFN/RBV/PI. Levels of mRNAs for selected ISGs and IFN-λs were evaluated by real-time PCR. All 31 patients with a favorable *IL28B* genotype and 13 of 19 with an unfavorable genotype achieved sustained virological responses (SVR). Levels of mRNA for *A20, SOCS1*, and *SOCS3*, known to suppress antiviral activity by interfering with the IFN signaling pathway, as well as *IRF1* were significantly higher at 8 h in patients with an unfavorable *IL28B* genotype than in those with a favorable one (*P* = 0.007, 0.026, 0.0004, 0.0006, respectively), especially in the non-SVR group. Particularly, the fold-change of *IRF1* at 8 h relative to baseline was significantly higher in non-SVR than in SVR cases with an unfavorable *IL28B* genotype (*P* = 0.035). In conclusion, levels of several mRNAs of genes suppressing antiviral activity in PBMCs during PEG-IFN/RBV/PI differed according to *IL28B* genotypes, paralleling treatment efficacy.

## Introduction

Chronic hepatitis C virus (HCV) infection is a significant risk factor for progressive liver fibrosis and hepatocellular carcinoma (HCC). Antiviral treatment improves the natural course in chronic hepatitis C (CHC) [1, 2]. Newly-developed treatments involving direct-acting antivirals (DAAs), including nonstructural (NS) 3/4A protease inhibitors have shown promising outcomes in combination with pegylated interferon (PEG-IFN) plus ribavirin (RBV) in several clinical trials. Thus >70% of patients infected with HCV genotype 1 are reported to achieve sustained virological responses (SVR) [3–5].

Recent genome-wide association studies (GWAS), including our own study on HCV infection [6], have identified a single nucleotide polymorphism (SNP) near the interleukin-28B (*IL28B*) gene encoding type III IFN-λ3 that was strongly associated with the response to PEG-IFN/RBV therapy for chronic HCV genotype 1 infection [6–9]. Furthermore, a recent meta-analysis showed that the *IL28B* genotype was also associated with efficacy of PEG-IFN/RBV plus NS3/4A protease inhibitor (PI) treatment, including telaprevir or boceprevir [10]. However, it is not known how the *IL28B* gene influences the elimination of HCV.

IFNs mediate their potent antiviral effects through the regulation of hundreds of IFN-stimulated genes (ISGs). Type I and III IFNs induce the transcription of ISGs by activating the Janus kinase-signal transducer and activator of transcription (Jak-STAT) pathway through different cell surface receptors [11–14]. Because it has been reported that a high level of expression of intrahepatic ISGs at baseline affects responses to PEG-IFN/RBV therapy [15, 16], several groups have investigated an association between *IL28B* genotype and the expression of intrahepatic ISGs [17, 18]. In addition, intrahepatic expression of genes involved in innate immunity, Toll-like receptor 3 (TLR3) and retinoic acid-inducible gene I (RIG-I) which are important in signaling pathways for IFN-β induction, were also associated with the *IL28B* genotype and response to PEG-IFN/RBV [19]. Nevertheless, we cannot fully explain treatment outcome by evaluating *IL28B* genotypes and measuring intrahepatic gene expressions at baseline. Changes of intrahepatic gene expressions cannot easily be evaluated due to the risk of complications caused by taking a liver biopsy. For this reason, several groups have assessed the response of ISGs to PEG-IFN/RBV using peripheral blood mononuclear cells (PBMCs) as a surrogate. However, most of these earlier studies found less marked correlations between the expression of ISGs in PBMCs and treatment efficacy or *IL28B* genotype, relative to what was seen in the liver of the same patients [15, 20, 21]. We also analyzed the expression of ISGs, which included previously reported genes [17, 19–21], in PBMCs during PEG-IFN/RBV therapy, indicating that several ISGs that suppressed the antiviral state by interfering with the IFN signaling pathway were associated with the *IL28B* genotype or response to PEG-IFN/RBV therapy. These included *A20*, suppressor of cytokine signaling 1 (*SOCS1*), *SOCS3*. In PEG-IFN/RBV/PI therapy, the expression of ISGs, IFN-λs, and molecules related to the innate immune system is expected to be changed greatly soon after the start of therapy, due to the effects of the PI. Hence, we prospectively collected PBMCs of patients treated with PEG-IFN/RBV/PI, and then evaluated associations between the levels of mRNAs for the selected ISGs or IFN-λs and the *IL28B* genotype or patient´s response to treatment.

## Patients and Methods

### Patients and treatment protocol

We prospectively enrolled a total of 50 CHC individuals infected with HCV genotype 1 who were treated with PEG-IFN/RBV/PI at Nagoya City University Hospital; 32 patients received telaprevir and 18 faldaprevir. All patients had tested positive for HCV RNA for more than 6 months. Patients chronically infected with hepatitis B virus or human immunodeficiency virus, or with other liver diseases such as autoimmune hepatitis and primary biliary cirrhosis, were excluded from this study.

The regimen of PEG-IFN/RBV/telaprevir therapy was as follows: PEG-IFN-α2b (1.5 μg/kg body weight subcutaneously once a week), RBV (600–1000 mg daily according to body weight), and telaprevir (standard dose of 2250 mg daily given three times a day every 8 hours or reduced dose of 1500 mg daily given twice a day every 12 hours) for 12 weeks, followed by an additional 12 weeks of PEG-IFN/RBV. In several patients, the initial dose of telaprevir was reduced to 1500 mg daily according to age, body weight, gender, or baseline hemoglobin level, at the discretion of the attending physicians. When marked adverse effects such as anorexia, anemia, neutropenia, thrombocytopenia, renal dysfunction or skin rash, developed, the dose of telaprevir was reduced to 1500 mg daily, and that of PEG-IFN or RBV was reduced according to the recommendation on the package inserts or the clinical condition of individual patients. The regimen of PEG-IFN/RBV/faldaprevir was as follows: PEG-IFN-α2a (180 μg subcutaneously once a week), RBV (600–1000 mg daily according to body weight), and faldaprevir (120 or 240 mg once-daily) for 12 or 24 weeks, followed by an additional PEG-IFN/RBV, making a total of 24 or 48 weeks. When marked adverse effects developed, the dose of PEG-IFN or RBV was reduced as mentioned above.

Written informed consent was obtained from each patient and the study protocol conformed to the ethics guidelines of the Declaration of Helsinki and was approved by the ethics review committees of Nagoya City University Hospital.

### Definition of virological response to treatment

Treatment outcomes were defined as SVR (undetectable HCV RNA levels 24 weeks after cessation of treatment), transient virological response (TVR; HCV RNA levels became undetectable during treatment but reappeared after the end of treatment), and non-virological response (NVR; HCV RNA levels never became undetectable).

### Detection of HCV RNA

Blood samples were obtained before treatment, and at week 1, 2, 4, 8, 12, and every 4 weeks up to treatment completion, and hematologic tests, blood chemistry and HCV RNA assays were performed. Follow-up measurements were obtained at week 4, 12 and 24 weeks after the end of treatment. HCV RNA levels were measured throughout the course of therapy using the COBAS TaqMan HCV test (Roche Diagnostics K.K., Tokyo, Japan). The measurement range of this assay is 1.2–7.8 log IU/mL.

### SNP genotyping

Genetic polymorphisms in SNPs of the *IL28B* gene (rs8099917) were determined according to the manufacturer´s instructions using TaqMan SNP Genotyping Assays and an ABI PRISM 7900HT Fast Real-Time PCR System (Applied Biosystems, Carlsbad, CA).

### Measurement of gene expression in PBMCs

Blood samples were collected from the patients at baseline, 8, and 24 hours (h) after the initial administration of PEG-IFN/RBV/PI. PBMCs were isolated from blood by Ficoll gradient centrifugation. Total RNA was extracted from PBMCs using the RNeasy Mini Kit (Qiagen, Valencia, CA). Complementary DNA (cDNA) synthesis was performed using 1.0 μg of total RNA isolated from PBMCs using the High Capacity RNA-to-cDNA kit (Applied Biosystems, Carlsbad, CA). Based on the recent studies [17, 19–21] as well as our analysis using an oligonucleotide DNA chip, Genopal (Mitsubishi Rayon CO., LTD. Tokyo, Japan) which can detect 208 genes related to innate immune responses (data not shown), we selected the following ISGs and IFN-λs: *ISG15, A20, zc3h12a*, ring finger protein 125 (*RNF125*), myxovirus resistance protein A (*MxA*), *IL1β, IL10*, interferon regulatory transcription factor 1 (*IRF1*), *SOCS1, SOCS2, SOCS3*, 2'-5'-oligoadenylate synthetase 1 (*OAS1*), double stranded RNA-dependent protein kinase (*PKR*), *IL28A, IL28B*, and *IL29*. We then quantified their mRNA levels by real-time detection polymerase chain reaction (PCR). The primers and probes for *IL28A* and *IL28B* were designed according to the previous report [22], and those of other genes were obtained from Applied Biosystems (Carlsbad, CA) as TaqMan Gene Expression Assays (Table A in S1 File). Amplification and detection were carried out using an ABI PRISM 7900HT Fast Real-Time PCR System (Applied Biosystems, Carlsbad, CA). Levels of mRNAs for ISGs were normalized against glyceraldehyde 3-phosphate dehydrogenase (GAPDH) as the internal control, and those for IFN-λs were measured using the calibration curves for each cDNA clone.

### Statistical Analysis

Categorical variables were compared between groups by the *χ* ^2^-test or Fisher’s exact test, and non-categorical variables by the Mann-Whitney U test. Correlations between continuous variables were analyzed using Pearson’s correlation coefficient test. *P* <0.05 was considered significant in all tests.

## Results

### Patient characteristics and distribution of *IL28B* genetic variants

The baseline clinical characteristics of the study population are described in Table 1. The unfavorable *IL28B* genotype, TG or GG (TG/GG) at rs8099917 was possessed by 38% (19/50) of the patients. Fourteen patients were treatment-naive. Of the 32 patients previously treated with PEG-IFN/RBV, 19 and 13 had TVR and NVR, respectively. Of the 13 NVR patients, 4 were null responders, defined as having an HCV RNA decrease of < 2 log IU/mL at week 12 after the start of therapy, relative to baseline. In addition, the clinical characteristics of the subsets of patients receiving telaprevir or faldaprevir are described in Table B in S1 File. The proportions of patients with an unfavorable *IL28B* genotype and NVR on prior PEG-IFN/RBV therapy were higher in patients who received faldaprevir.

**Table 1:** Baseline clinical characteristics of the 50 chronic hepatitis C patients treated with PEG-IFN, RBV and protease inhibitor.

Characteristic	(n = 50)
Male gender	30 (60%)
Age, years	55 (29–70)
Hemoglobin, g/dL	14.8 (12.0–17.1)
Platelet count, ×10^4^ /μL	16.2 (9.8–27.9)
ALT, IU/L	34 (13–212)
γ-GTP, IU/L	28 (12–258)
HCV RNA, log IU/ml	6.7 (4.8–7.5)
rs8099917, TT / TG / GG	31 / 16 / 3
Fibrosis stage, F0 / 1 / 2 / 3 / 4 / N.D.	5 / 20 / 6 / 3 / 1 / 15
Prior treatment	
naïve / IFN mono / IFN +RBV / PEG-IFN+RBV	14 / 2 / 2 / 32
Treatment efficacy of PEG-IFN+RBV, TVR / NVR	19 / 13

Abbreviations: ALT, alanine aminotransferase; γ-GTP, γ-glutamyl transpeptidase; N.D., not determined; IFN, interferon; RBV, ribavirin; PEG-IFN, pegylated interferon; TVR, transient virological response; NVR, non-virological response.

rs8099917: TT is favorable for treatment efficacy.

Data are expressed as numbers for categorical data or the median (range) for continuous data.

All 31 patients with a favorable *IL28B* genotype and 13 of 19 with an unfavorable genotype achieved SVR on PEG-IFN/RBV/PI treatment. Hence, the total SVR rate was 88% (44/50). The detailed information of the six non-SVR cases are described as follows: one patient did not response to PEG-IFN/RBV/telaprevir up to week 12 (quantity of HCV RNA at week 12 was 3.7 log IU/mL) and the therapy was discontinued; three had virological breakthrough at week 17, 38, 40 during PEG-IFN/RBV/faldaprevir and the therapies were discontinued; two were relapsed after the completion of PEG-IFN/RBV/faldaprevir. Thus these six patients resulted in non-SVR, though they were given enough doses of drugs. In four SVR cases, the therapies were discontinued at week 9, 11, 18, 20 during PEG-IFN/RBV/telaprevir due to adverse events. Other clinical characteristics of the patients according to *IL28B* genotype and treatment efficacy are described in Table C in S1 File.

### Gene expression of ISGs and IFN-λs induced by PEG-IFN/RBV/PI in patients stratified according to *IL28B* genotype

**Fig 1:**
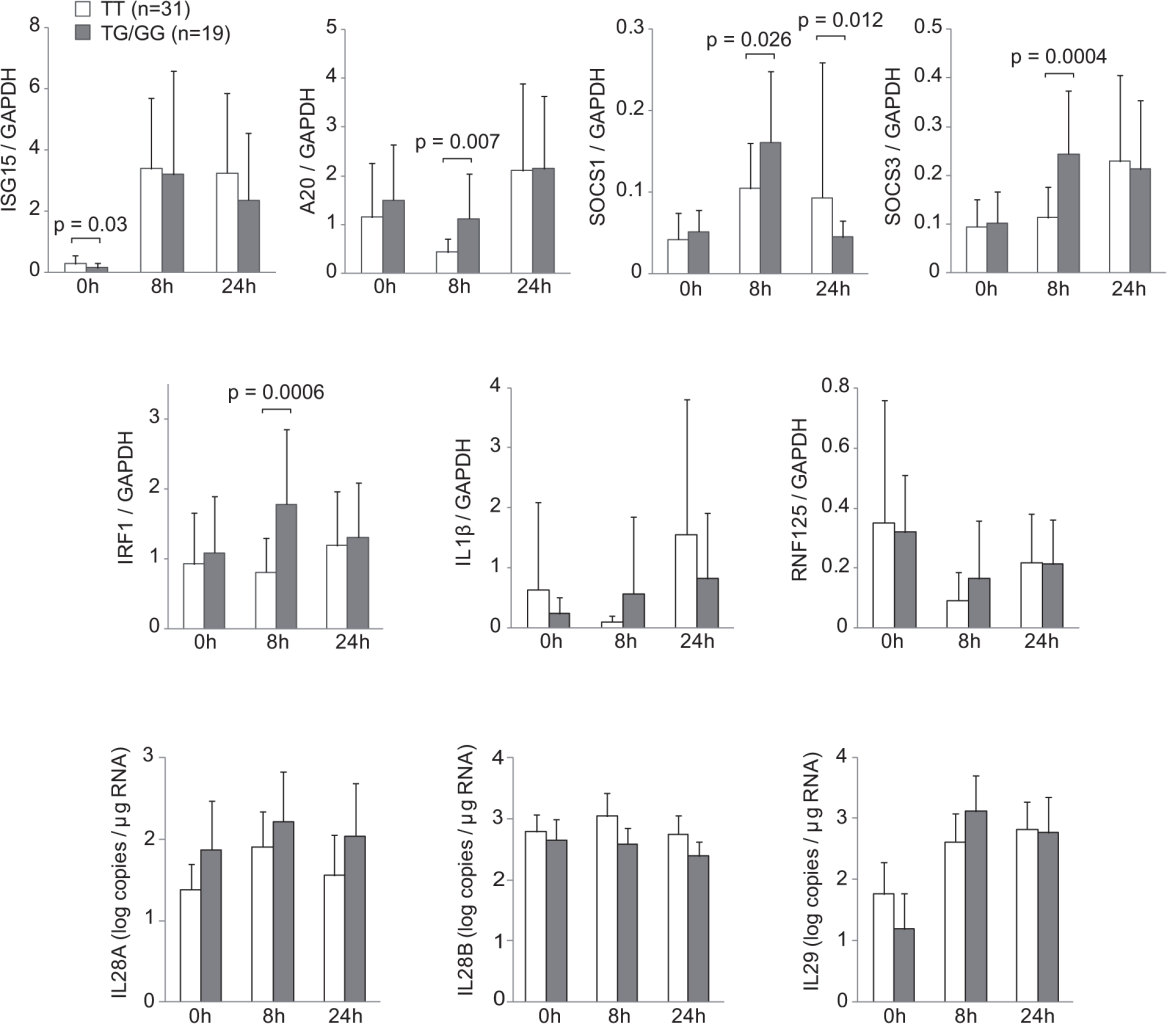
Expression of interferon-stimulated genes (ISGs) and interferon-lambdas (IFN-λs) in peripheral blood mononuclear cells at baseline, 8, and 24 hours after the initial administration of pegylated interferon, ribavirin, plus NS3/4A protease inhibitor, in patients stratified according to *IL28B* genotype. Levels of mRNAs for ISGs were normalized against glyceraldehyde 3-phosphate dehydrogenase (GAPDH), and those for IFN-λs were measured using the calibration curves for each cDNA clone. Bars and error bars represent means and standard deviations, respectively. TT and TG/GG at rs8099917 is a favorable and an unfavorable *IL28B* genotype for treatment responses, respectively.

**Fig 2:**
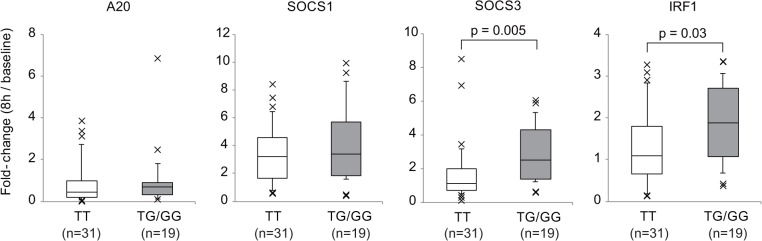
Fold-changes of mRNAs including suppressive genes in PBMCs at 8 hours relative to baseline in patients stratified according to *IL28B* genotype. TT and TG/GG at rs8099917 is a favorable and an unfavorable *IL28B* genotype for treatment responses, respectively. Boxes represent the interquartile range of the data. The lines across the boxes and the numbers indicate the median values. The hash marks above and below the boxes indicate the 90th and 10th percentiles for each group, respectively.

Eight hours after the initial administration of PEG-IFN/RBV/PI, levels of mRNAs for *A20, SOCS1*, and *SOCS3* known to be genes suppressing antiviral activity via the IFN signaling pathway, as well as *IRF1* were found to be significantly higher in patients with TG/GG at rs8099917, an unfavorable *IL28B* genotype (*P* = 0.007, 0.026, 0.0004, and 0.0006, respectively). In contrast, the levels of mRNAs for *IL28A, IL28B*, and *IL29* were not different regardless of the *IL28B* genotype, although the expression of *IL28B* itself tended to be higher in patients with a favorable *IL28B* type (Fig. 1). There were also no significant differences in the levels of other mRNAs for *ISG15, IL1β, RNF125* (Fig. 1), *zc3h12a, MxA, IL10, SOCS2, OAS1* or *PKR* at 8 h (data not shown). We analyzed changes in expression of the genes for *A20, SOCS1, SOCS3* and *IRF1* between baseline and 8 h and found that the fold-changes of *SOCS3* and *IRF1* were significantly higher in patients with an unfavorable *IL28B* genotype (*P* = 0.005 and 0.030, respectively) (Fig. 2).

### Correlations of gene expression in PEG-IFN/RBV/PI treatment

**Fig 3:**
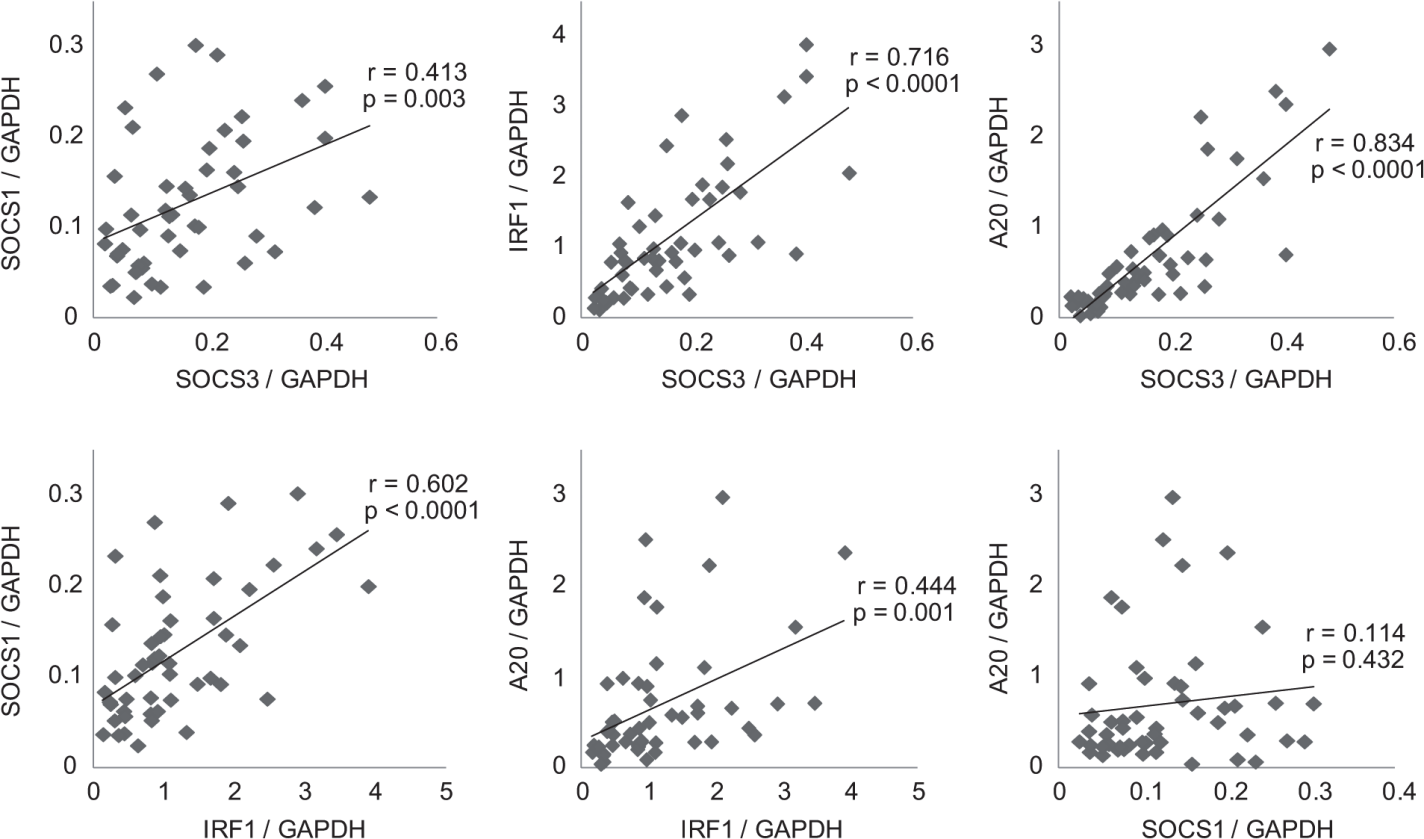
Relationships between levels of mRNAs including suppressive genes in PBMCs 8 hours after the initial administration of pegylated interferon, ribavirin, plus NS3/4A protease inhibitor in all patients. Levels of mRNAs including suppressive genes were normalized against glyceraldehyde 3-phosphate dehydrogenase (GAPDH).

We evaluated the correlations of the levels of mRNAs for genes implicated in suppressing the antiviral state each other and with those promoting it, (*ISG15* and *IL28B*), in all 50 cases. The expression levels of most of the mRNAs for suppressive genes such as *A20, SOCS1* and *SOCS3*, as well as *IRF1* were significantly correlated with each other at 8 h (Fig. 3) as well as at baseline (Figure A in S1 File) and 24h (Figure B in S1 File). However, they did not correlate with those of *ISG15* and *IL28B* at 8 h (Figure C in S1 File) as well as at baseline and 24h (data not shown).

### Associations between ISGs including suppressive genes against the antiviral state and prediction of treatment efficacy

**Fig 4:**
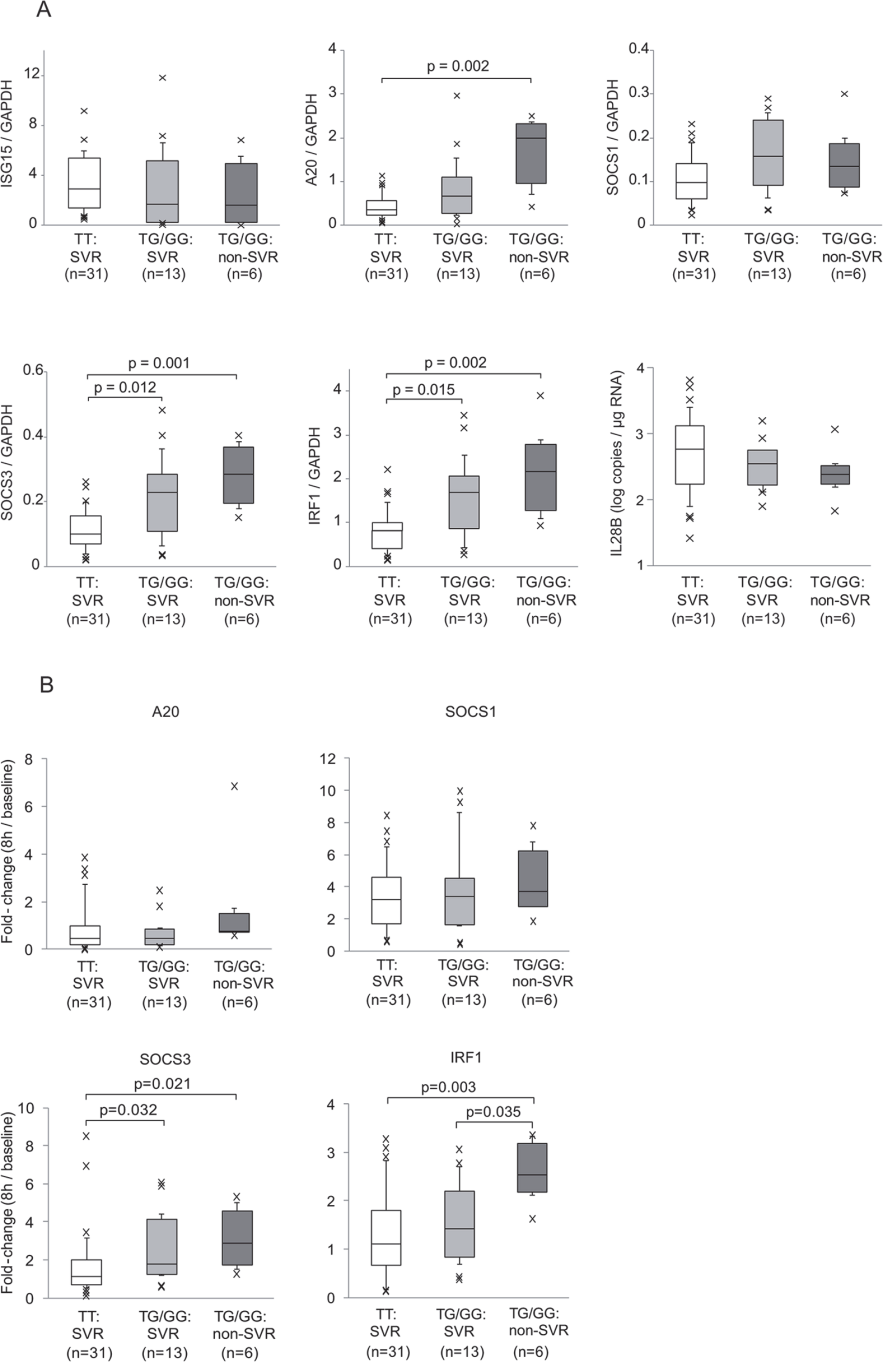
Associations between the expression of ISGs or IFN-λ3 and treatment efficacy. Patients were divided into three groups according to *IL28B* genotype at rs8099917 and treatment outcome: TT; SVR (n = 31), TG/GG; SVR (n = 13), and TG/GG; non-SVR (n = 6). (A) Expression of *ISG15, IL28B* and suppressive genes in PBMCs at 8 hours after the initial administration of pegylated interferon, ribavirin, plus NS3/4A protease inhibitor in each group. (B) Fold-changes of mRNAs including suppressive genes at 8 hours relative to baseline in each group. Levels of mRNAs including suppressive genes and *ISG15* were normalized against glyceraldehyde 3-phosphate dehydrogenase (GAPDH), and those for *IL28B* were measured using the calibration curves of cDNA clone. TT and TG/GG at rs8099917 is a favorable and an unfavorable *IL28B* genotype for treatment responses, respectively. Boxes represent the interquartile range of the data. The lines across the boxes and the numbers indicate the median values. The hash marks above and below the boxes indicate the 90th and 10th percentiles for each group, respectively.

To examine the association between the expression of genes suppressing antiviral activity and treatment efficacy, we divided the patients into three groups according to *IL28B* genotype and treatment outcome, as follows: TT: SVR (n = 31); TG/GG: SVR (n = 13); TG/GG: non-SVR (n = 6) (Table C in S1 File). We then compared the levels of mRNAs for *A20, SOCS1, SOCS3, IRF1, ISG15*, and *IL28B* among the groups. We found that the levels of mRNAs for *A20, SOCS3* and *IRF1* at 8 h were significantly higher in TG/GG: non-SVR than in TT: SVR (*P* = 0.002, 0.001, and 0.002, respectively). Moreover, the levels of mRNAs for *SOCS3* and *IRF1* were also higher in TG/GG: SVR than in TT: SVR (*P* = 0.012 and 0.015, respectively) (Fig. 4A). Whereas the level of mRNA for *IL28B* tended to be higher in the order TT: SVR, TG/GG: SVR, TG/GG: non-SVR, there were no significant differences among the three groups. Although we also compared the expression levels of these genes at baseline and 24h among the same three groups, we could not find the definite tendency (data not shown). Next, we analyzed the changes in expression of *A20, SOCS1, SOCS3*, and *IRF1* from baseline to 8 h and found that the fold-change of *IRF1* was significantly higher in TG/GG: non-SVR than in TG/GG: SVR as well as in TT: SVR (*P* = 0.035 and 0.003, respectively). Similarly, the fold-change of *SOCS3* was higher in TG/GG: non-SVR and SVR than in TT: SVR (*P* = 0.021 and 0.032, respectively) (Fig. 4B). Collectively, one can conclude that levels of expression of mRNAs including these suppressive genes early after the initial administration of PEG-IFN/RBV/PI were different in patients with different *IL28B* genotypes and different treatment efficacies.

## Discussion

In the present study, we determined that mRNAs for *A20, SOCS1* and *SOCS3*, known to be genes suppressing antiviral activity via the IFN signaling pathway, as well as *IRF1* were highly expressed in PBMCs early after the initial administration of PEG-IFN/RBV/PI in patients with an unfavorable *IL28B* genotype, especially the non-SVR group. The correlations of mRNA expression levels of these genes, *ISG15*, and *IL28B* suggest that the expression levels of these suppressive genes show similar dynamics independently with the genes promoting the antiviral state in the interferon signaling pathway. Asahina *et al*. showed that the induction of several ISGs in PBMCs after the initial administration of PEG-IFN/RBV tended to be stronger in SVR than in NVR, but in their study the difference was not statistically significant [20]. The HCV NS3/4A protease cleaves and inactivates two important signaling molecules in the innate immune system, the mitochondrial antiviral signaling protein (MAVS), an essential component of the RIG-I pathway [23], and the Toll-IL-1 receptor domain-containing adaptor inducing IFN-β (TRIF), an adaptor in the TLR3 pathway [24]. Because PI inhibits the function of NS3/4A protease, it is expected to affect these pathways and the expression of ISGs. Indeed, Kalkeri *et al*. showed that PIs including telaprevir, boceprevir, and simeprevir can restore innate immunity by directly inhibiting NS3/4A protease-mediated cleavage of MAVS at clinically achievement concentrations *in vitro* using HCV replicon cells [25]. Therefore, in PEG-IFN/RBV/PI therapy, the expression of ISGs, IFN-λs, and molecules related to the innate immune system may be more markedly altered early after the start of this therapy than PEG-IFN/RBV therapy without PI. This may be the reason why we were able to determine the differences of expression of these suppressive gene mRNAs. We preliminarily compared the mRNA levels of the suppressive genes, *ISG15, MAVS* and *TRIF* in PBMCs between in patients of this study (data of two patients were unavailable) and in those with PEG-IFN/RBV therapy, whose characteristics are described in Table D in S1 File. There were no differences for these genes at 8h/baseline, however, the inductions of mRNA for several genes such as *A20, IRF1, SOCS3*, and *MAVS* at 24h/baseline were greater in PEG-IFN/RBV/PI (Figure D in S1 File). In general, previous studies have shown that HCV mainly could replicate in liver and lympho-trophic HCV would be minor, therefore it is not main event that HCV NS3/4A cleaves MAVS or TRIF in PBMCs. We speculate that inhibiting cleavages of MAVS and TRIF by PI in liver more strongly induces IRF3 activation and subsequent IFN-α/β and ISGs production, resulting in the activation of RIG-I, TLR3, and IFN signaling pathway in livers and PBMCs. For these reasons, we guess that the several genes related with these pathways were more strongly induced at 24 h in patients treated with PEG-IFN/RBV/PI. Further studies will be required to evaluate the effect of PI itself on the IFN signaling pathway in PBMCs or liver. In the present study, levels of mRNAs for IFN-λs as well as common ISGs promoting the antiviral state at baseline and during therapy were not found to be significantly associated with the *IL28B* genotype or treatment efficacy. Recently, Honda *et al*. showed that there was no difference of pretreatment mRNA expression of ISGs as well as *IL28A/B* in blood between *IL28B* genotypes or responses to PEG-IFN/RBV [26]. These results support our data at baseline. Interestingly, they also indicated that the expression of ISGs at baseline correlated significantly between liver and blood in patients with a favorable *IL28B* genotype, not in those with an unfavorable genotype [26].

As previously reported, *SOCS1* suppresses the Jak/STAT pathway, specifically STAT1 [27]. *SOCS3* inhibits expression of ISGs such as *OAS1* and *PKR* through inactivation of the Jak-STAT pathway [28]. *A20* is a suppressive factor of the nuclear factor-kappa B pathway [29] and a candidate negative regulator of the signaling cascade leading to *IRF3* activation in the innate antiviral response [30]. *IRF1* is well known as a transcription factor that activates the expression of *IFN-β*, leading to enhancement of IFN signaling [31, 32]. However, Moore *et al*. showed that *IRF1* enhances the expression of *SOCS1* using rat pancreatic β-cells, and suggested that *IRF1* provides a negative feedback on STAT1 and downstream signaling via STAT1 dephosphorylation by SOCS1 up-regulation [33]. Furthermore, in our preliminary *silico* analysis, *IRF1* is expected to bind the promoter region of *A20* (data not shown), and thus may influence the functional expression of *A20* through transactivation of *A20* promoter, resulting in negative regulation of IFN signaling cascade. Collectively, these suppressive factors may negatively affect the IFN signaling pathway and the production of ISGs or IFN in HCV infection. Abe *et al*. showed that pretreatment intrahepatic levels of two ISGs suppressing the antiviral state, *A20* and *Zc3h12a*, were significantly higher in patients with a favorable *IL28B* genotype, and that a high level of *SOCS1* was a predictive factor for NVR. In contrast, they found that levels of most of the ISGs promoting the antiviral state via the IFN signaling pathway and *IL28* were significantly lower in patients with a favorable *IL28B* genotype [34]. Thus, the expression of these suppressive genes in the liver might influence treatment efficacy. Taking this previous report together with our results using PBMCs presented here, we may conclude that the levels of mRNAs for suppressive genes in liver and PBMCs are associated with *IL28B* polymorphisms.

The mechanism of interaction between IFN-λ and ISG expression in liver or PBMC resulting in the elimination of HCV has not yet been elucidated. Using primary hepatocytes from humans and chimpanzees, Thomas *et al*. found that type III but not type I IFNs are primarily induced after HCV infection, and that their degree of induction is closely correlated with the levels of ISGs [35]. These results strongly suggest that hepatic IFN-λ production may have important roles and could be a principal driver of ISG induction in response to HCV infection. On the other hand, in a chronically HCV-infected chimeric mouse model, larger amounts of IFN-λs were produced by HCV-infected human hepatocytes with a favorable *IL28B* genotype on treatment with IFN-α [36]. Recently, it has been shown in *ex vivo* experiments that a certain subset of dendritic cells (DCs) within human PBMCs recognized HCV and produced large amounts of IFN-λs [37, 38], and that the capacity for producing IFN-λ3 was superior in subjects with a favorable *IL28B* genotype [38]. Furthermore, IFN-α directly affected DC function and significantly increased IFN-λ production [37]. These findings suggest that in addition to HCV-infected hepatocytes, DCs within PBMCs may play a crucial role in the response to IFN treatment via production of IFN-λs and ISGs. We speculate that the levels of several suppressive ISGs in liver and DCs might be different according to the *IL28B* genotype, implying a difference of response to treatment. In addition, it has not been fully elucidated how IFN-λs or ISGs influence effector cells such as natural killer (NK) cells or cytotoxic T lymphocytes in HCV infection. Although we also investigated several cytokines such as IL-2, 4, 5, 6, 10, 12, IFN-γ, and tumor necrosis factor (TNF)-α in patients´ serum during PEG-IFN/RBV/PI, we did not find any differences attributable to *IL28B* genotype or any associating with treatment efficacy (data not shown). Intriguingly, recent study showed that infiltration of various immune cells including DCs, NK cells, and T cells, and expression of various chemokines in liver were repressed in patients with an unfavorable *IL28B* genotype, and their up-regulation of intrahepatic ISGs was mediated by multiple factors, including *IL28A/B*, IFN-λ4, and wingless-related MMTV integration site 5A [26]. Further studies will be required to identify the role of ISGs suppressing the antiviral state in hepatocytes or DCs, and how IFN and ISGs effect the elimination of HCV.

This study has several limitations. The treatment regimens were different for different patients, including the type of PI, its dose, and duration of therapy, especially in the patients receiving faldaprevir, even though faldaprevir dose and treatment duration reportedly had little influence on SVR rates in some clinical trials [39]. Furthermore, there was bias in that the proportion of intractable cases was higher in the patients receiving faldaprevir. Second, the number of analyzed cases was small, especially the non-SVR cases. Third, we analyzed the expression of the selected genes in PBMCs at baseline and the only early periods after the initial administration of PEG-IFN/RBV/PI. Further comprehensive gene expression analysis including more prolonged kinetics of genes are necessary in a large number of patients treated with the same regimen to verify the results of the present study.

The findings in this study contribute to our understanding of immune response to HCV during PEG-IFN/RBV/PI therapy. IFN-free therapy is expected to be useful especially in IFN-resistant patients and may become the standard of care in the near future. Future study should evaluate immune responses under IFN-free therapy as well as IFN-based therapy to clarify the mechanism of HCV elimination.

In conclusion, the expression of several genes, which suppress antiviral activity by interfering IFN signaling pathway, in PBMCs during PEG-IFN/RBV/PI was found to be different according to the patient´s *IL28B* genotype and treatment response.

## Supporting Information

S1 File Table A, Primers and probes for quantitative real-time PCR of ISGs and IFN-λs. **Table B,** Clinical characteristics of chronic hepatitis C patients treated with PEG-IFN/RBV plus telaprevir or faldaprevir. **Table C,** Clinical characteristics of chronic hepatitis C patients according to *IL28B* genotype and treatment efficacy. **Table D,** Clinical characteristics of chronic hepatitis C patients treated with PEG-IFN/RBV. **Figure A,** Correlations between levels of mRNAs including suppressive genes at baseline. **Figure B,** Correlations between levels of mRNAs including suppressive genes at 24 hours after the initial administration PEG-IFN, RBV, plus NS3/4A protease inhibitor. **Figure C,** Correlation between levels of mRNA including suppressive genes and those for *IL28B* or *ISG15* at 8 hours after the initial administration PEG-IFN, RBV, plus NS3/4A protease inhibitor. **Figure D,** Fold-changes of mRNAs for ISGs, *TRIF* and *MAVS* in PBMCs at 8, 24 hours relative to baseline in PEG-IFN/RBV and PEG-IFN/RBV/PI therapy.
(PDF)
