# Supplementary material for: Influence of Genes Suppressing Interferon Effects in Peripheral Blood Mononuclear Cells during Triple Antiviral Therapy for Chronic Hepatitis C
Source: PLoS One. 2015 Feb 23;10(2):e0118000. doi: 10.1371/journal.pone.0118000 (PMC4338062; doi:10.1371/journal.pone.0118000)
Supplement: S1 File — Table B, Clinical characteristics of chronic hepatitis C patients treated with PEG-IFN/RBV plus telaprevir or faldaprevir. Table C, Clinical characteristics of chronic hepatitis C patients according to IL28B genotype and treatment efficacy. Table D, Clinical characteristics of chronic hepatitis C patients treated with PEG-IFN/RBV. Figure A, Correlations between levels of mRNAs including suppressive genes at baseline. Figure B, Correlations between levels of mRNAs including suppressive genes at 24 hours after the initial administration PEG-IFN, RBV, plus NS3/4A protease inhibitor. Figure C, Correlation between levels of mRNA including suppressive genes and those for IL28B or ISG15 at 8 hours after the initial administration PEG-IFN, RBV, plus NS3/4A protease inhibitor. Figure D, Fold-changes of mRNAs for ISGs, TRIF and MAVS in PBMCs at 8, 24 hours relative to baseline in PEG-IFN/RBV and PEG-IFN/RBV/PI therapy. (PDF) [file pone.0118000.s001.pdf]

Supplementary Information

Influence of genes suppressing interferon effects in peripheral blood mononuclear cells during triple antiviral therapy for chronic hepatitis C (published by *PLOS ONE*)

Sayuki Iijima, Kentaro Matsuura, Tsunamasa Watanabe, Koji Onomoto, Takashi Fujita, Kyoko Ito, Etsuko Iio, Tomokatsu Miyaki, Kei Fujiwara, Noboru Shinkai, Atsunori Kusakabe, Mio Endo, Shunsuke Nojiri, Takashi Joh, and Yasuhito Tanaka*

* Corresponding author:

Prof. Yasuhito Tanaka, M.D., Ph.D.,

Department of Virology, Liver Unit,

Nagoya City University Graduate School of Medical Sciences,

E-mail: ytanaka@med.nagoya-cu.ac.jp

Table A. Primers and probes for quantitative real-time PCR of ISGs and IFN-λs. TaqMan Gene Expression Assay primer and probe sets (Applied Biosystems) and primer and probe set for *IL28A*, *IL28B* genes are listed.

GAPDH	Hs02758991_g1
ISG15	Hs01921425_s1
IRF1	Hs00971960_m1
A20	Hs00234713_m1
SOCS1	Hs00705164_s1
SOCS3	Hs02330328_s1
RNF125	Hs00215201_m1
IL-10	Hs00961622_m1
IL-1β	Hs01555409_g1
IL-28A	hIL28A-S: 5’-GCTGAAGGACTGCAGGTGCCA-3’<br>hIL28A-R:5’- GGGCTGGTCCAAGACGTCCA -3’<br>hIL28A-probe: 5’-ATGGCTTTGGAGGCTGAGCTGGCC-3
IL-28B	hIL28B-S: 5’-GCTGAAGGACTGCAAGTGCCG-3’<br>hIL28B-R: 5’-GGGGCTGGTCCAAGACATCCC-3’<br>hIL28B-probe: 5’- CGGGGCGCTCCCTCACCTGC-3’
IL-29	Hs00601677_g1

Table B. Clinical characteristics of chronic hepatitis C patients treated with PEG-IFN/RBV plus telaprevir or faldaprevir.

Characteristic	Telaprevir (n=32)	Faldaprevir (n=18)
Male gender	21 (66%)	9 (50%)
Age, years	55 (29 - 70)	54 (33 - 68)
Hemoglobin, g/dL	14.8 (12.0 - 16.7)	14.6 (12.1 - 17.1)
Platelet count, $\times 10^4$ /μL	14.3 (9.8 - 27.9)	19.6 (9.9 - 27.2)
ALT, IU/L	35 (13 - 212)	33 (15 - 100)
γ-GTP, IU/L	28 (12 -258)	26 (12 - 103)
HCV RNA, log IU/ml	6.8 (4.8 - 7.5)	6.6 (5.5 - 7.3)
rs8099917, TT / TG / GG	26 / 5 / 1	5 / 11 / 2
Fibrosis stage, F0 / 1 / 2 / 3 / 4 / N.D.	2 / 9 / 4 / 2 / 1 / 14	3 / 11 / 2 / 1 / 0 / 1
Prior treatment		
naïve / IFN mono / IFN +RBV / PEG-IFN+RBV	8 / 2 / 2 / 20	6 / 0 / 0 / 12
Treatment efficacy of PEG-IFN+RBV, TVR / NVR	17 / 3	2 / 10

Abbreviations: ALT, alanine aminotransferase; γ-GTP, γ-glutamyl transpeptidase; N.D., not determined; IFN, interferon; RBV, ribavirin; PEG-IFN, pegylated interferon; TVR, transient virological response; NVR, non-virological response.
rs8099917 : TT is favorable for treatment efficacy.

Data are expressed as number for categorical data or the median (range) for continuous data.

Table C. Clinical characteristics of chronic hepatitis C patients according to *IL28B* genotype and treatment efficacy.

Characteristic	TT: SVR (n = 31)	TG/GG: SVR (n = 13)	TG/GG: non-SVR (n = 6)
Male gender	19 (61%)	6 (46%)	5 (83%)
Age, years	55 (29 - 69)	59 (32 - 70)	50 (44 - 67)
Hemoglobin, g/dL	14.4 (12.0 - 16.7)	14.5 (12.1 - 16.2)	15.7 (15.2 - 17.1)
Platelet count, $\times 10^{4}$/µL	14.3 (10.1 - 27.2)	17.8 (9.8 - 27.9)	21.0 (9.9 - 25.4)
ALT, IU/L	36 (13 - 212)	27 (16 - 100)	67 (22 - 98)
γ-GTP, IU/L	27 (12 - 258)	28 (15 - 60)	40 (19 - 143)
HCV RNA, log IU/ml	6.8 (5.6 - 7.5)	6.0 (4.8 - 7.4)	6.9 (5.5 - 7.5)
Fibrosis stage, F0 / 1 / 2 / 3 / 4 / N.D.	3 / 7 / 4 / 2 / 1 / 14	2 / 9 / 1 / 1 / 0 / 0	0 / 4 / 1 / 0 / 0 / 1
Prior treatment			
naïve / IFN mono / IFN +RBV / PEG-IFN+RBV	9 / 2 / 2 / 18	3 / 0 / 0 / 10	2 / 0 / 0 / 4
Treatment efficacy of PEG-IFN+RBV, TVR / NVR	16 / 2	3 / 7	0 / 4
Present treatment			
Telaprevir / Faldaprevir, plus PEG-IFN/RBV	26 / 5	5 / 8	1 / 5

Abbreviations: SVR, sustained virological response; ALT, alanine aminotransferase; γ-GTP, γ-glutamyl transpeptidase; N.D., not determined; IFN, interferon; RBV, ribavirin; PEG-IFN, pegylated interferon; TVR, transient virological response; NVR, non-virological response.

rs8099917 : TT is favorable for treatment efficacy.

Data are expressed as number for categorical data or the median (range) for continuous data.

Table D. Clinical characteristics of chronic hepatitis C patients treated with PEG-IFN/RBV.

Characteristic	(n = 11)
Male gender	7 (64%)
Age, years	57 (36 - 68)
Hemoglobin, g/dL	14.2 (12.3 - 17.7)
Platelet count, $\times 10^4$/μL	14.0 (9.2 - 23.7)
ALT, IU/L	40 (23 - 205)
γ-GTP, IU/L	31 (16 - 176)
HCV RNA, log IU/ml	6.5 (4.8 - 7.2)
rs8099917, TT / TG / GG	7 / 4 / 0
Fibrosis stage, F0 / 1 / 2 / 3 / 4 / N.D.	1 / 6 / 3 / 0 / 0 / 1
Treatment efficacy	
SVR / Relapse	7 / 4

Abbreviations: ALT, alanine aminotransferase; γ-GTP, γ-glutamyl transpeptidase; N.D., not determined; RBV, ribavirin; PEG-IFN, pegylated interferon; SVR, sustained virological response.

rs8099917 : TT is favorable for treatment efficacy.

Data are expressed as numbers for categorical data or the median (range) for continuous data.

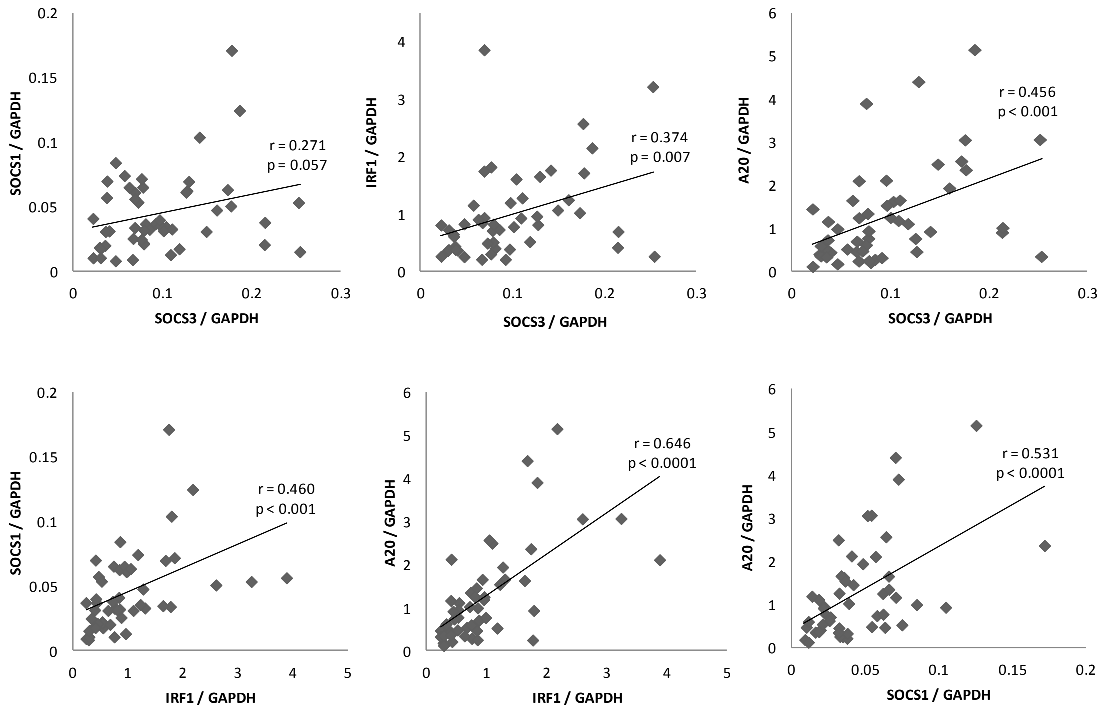

Figure A. Correlations between levels of mRNAs including suppressive genes at baseline.

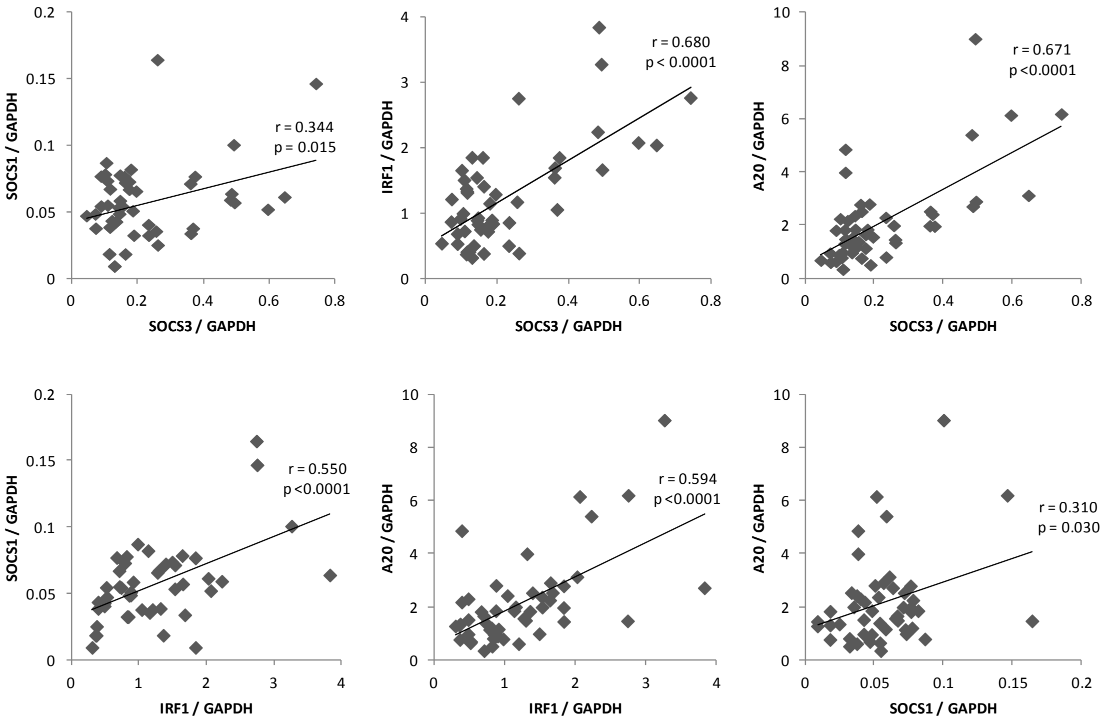

Figure B. Correlations between levels of mRNAs including suppressive genes at 24 hours after the initial administration PEG-IFN, RBV, plus NS3/4A protease inhibitor.

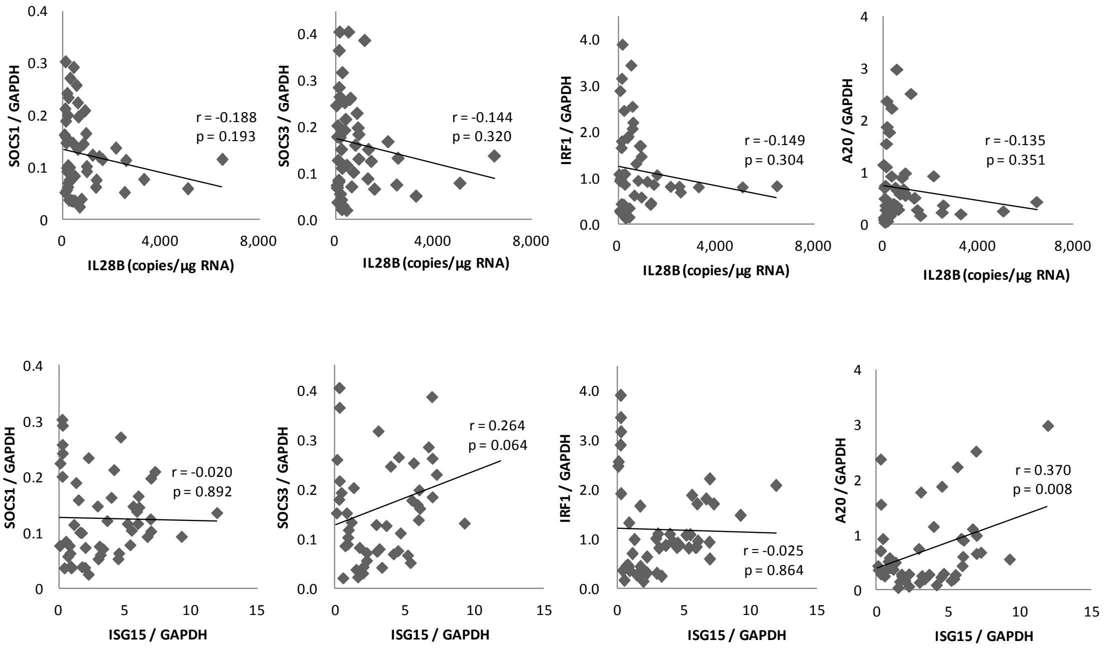

Figure C. Correlation between levels of mRNA including suppressive genes and those for *IL28B* or *ISG15* at 8 hours after the initial administration PEG-IFN, RBV, plus NS3/4A protease inhibitor.

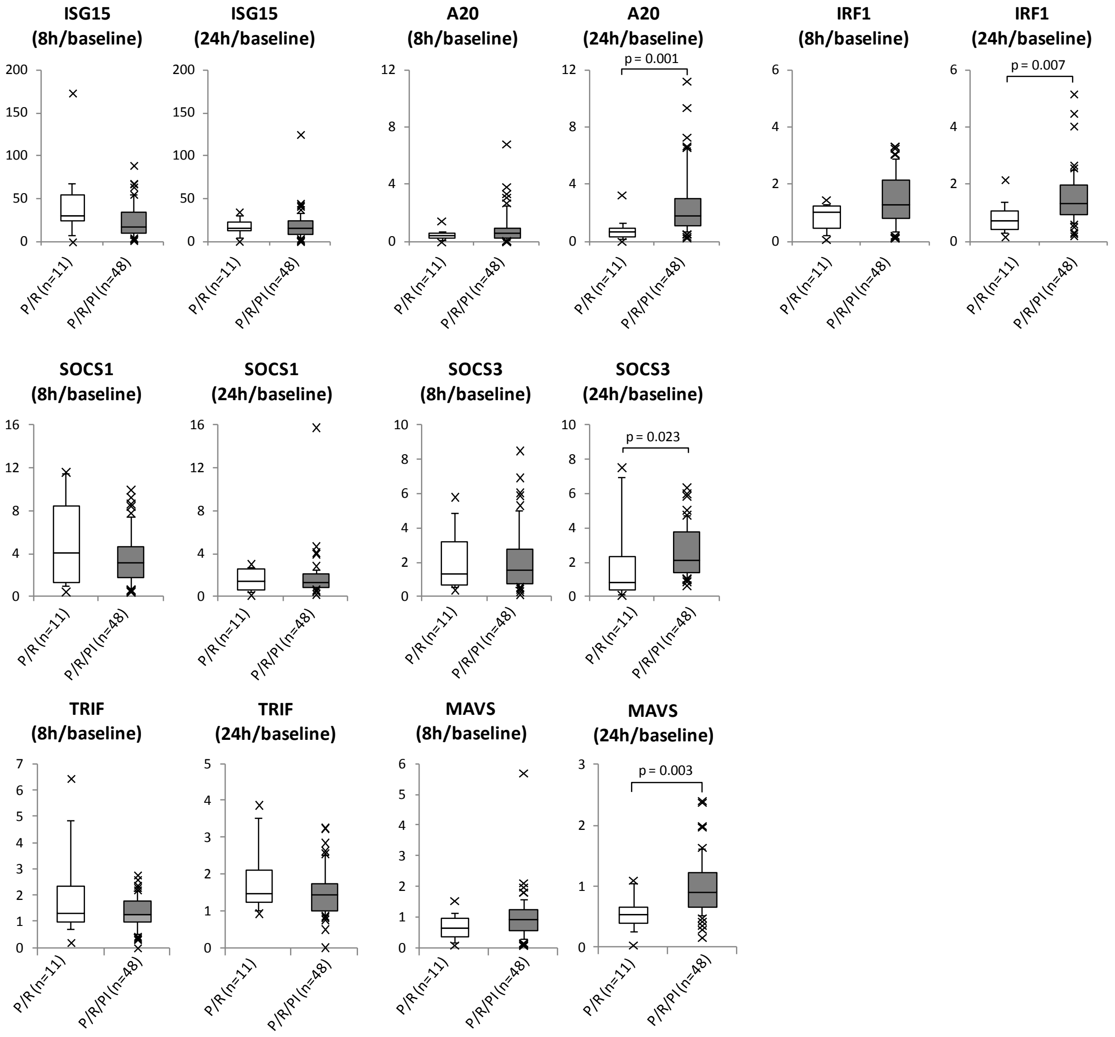

Figure D. Fold-changes of mRNAs for ISGs, *TRIF* and *MAVS* in PBMCs at 8, 24 hours relative to baseline in PEG-IFN/RBV and PEG-IFN/RBV/PI therapy.
